# The effects of endometrial injury on intrauterine insemination outcome: A randomized clinical trial

**Published:** 2014-09

**Authors:** Afsoon Zarei, Saeed Alborzi, Nasrin Dadras, Ghazal Azadi

**Affiliations:** *Department of Obstetrics and Gynecology, Shiraz University of Medical Sciences, Shiraz, Iran.*

**Keywords:** *Infertility*, *Female*, *Endometrial injury*, *Pregnancy*, *Intrauterine Insemination (IUI)*

## Abstract

**Background:** Implantation is considered as the rate-limiting step in success of assisted reproduction techniques, and intrauterine insemination cycles. It might be affected by ovarian superovulation and endometrial local scratching.

**Objective:** This study aims to investigate the effect of local endometrial injury on the outcome of IUI cycles.

**Materials and Methods: **In this randomized clinical trial 144 women with unexplained infertility, mild male factor, and mild endometriosis randomly divided into two study groups through block randomization. The patients were randomly assigned to undergo endometrial biopsy between days 6-8 of the previous menstrual cycle before IUI (n=72, IUI cycles =126) or receive no interventions (n=72, IUI cycles=105).

**Results: **The pregnancy rate per patient was 17 (23.6%) and 14 (19.4%) in endometrial biopsy and control groups, respectively (p=0.686). The pregnancy rate per cycle was 17/126 (13.5%) and 14/105 (13.3%) in endometrial biopsy and control groups, respectively (p=0.389). The abortion rate was comparable between the two groups (6.9% vs. 9.7%; p=0.764). The ongoing pregnancy rate was found to be comparable between the two study groups, as well (16.7% vs. 9.7%; p=0.325). Endometrial thickness (p=0.609) was comparable between the groups; however E_2_ was significantly lower in the endometrial biopsy group (p<0.001).

**Conclusion:** Application of local endometrial injury in the cycle before the IUI cycles is not associated with increased pregnancy rate per patient and per cycle, decreased abortion, and increased endometrial thickness.

## Introduction

The opposition and attachment processes of the embryo to the endometrium are the primary stages of implantation. Subsequently, the embryo invades to the stroma of the uterine wall. Implantation is considered to be a complex and multifactorial process involving several growth factors and cytokines which regulate the interaction between the embryo and the endometrium (1). It is proposed that approximately 75% of the embryos are lost at the time of implantation resulting in implantation failure (2).

Implantation is considered as the rate-limiting step not only in ART, including In Vitro Fertilization (IVF) and Intracytoplasmic Sperm Injection (ICSI), but also IUI cycles, which is affected by several biological and hormonal markers (3-5). Interactions between the endometrium and the embryo as well as endometrial receptivity are considered as two strong factors affecting the outcome of implantation (6). The receptivity of the endometrium is an estrogen- and progesterone-dependent phenomenon (7). The endometrial receptivity is proposed to be a period of time in which the endometrium becomes capable of receiving and adhering the human embryo (4, 7). 

This period of time is self-limited and is usually limited to the days 19-24 of the menstrual cycle in humans and can be affected by controlled ovarian hyperstimulation (COH) and endometrial local scratching (4-13). Animal studies have demonstrated that scratching and trauma to the endometrium provoke the decidualization and endometrial receptivity in animals (9). In this regards, Barash *et al* demonstrated that performing endometrial biopsy on days 8, 12, 21, and 26 of the menstrual cycle was associated with higher pregnancy rate after IVF (10). 

These results were further confirmed by Zhou *et al* who showed that inducing local injury to the endometrium in COH cycles was associated with a higher success rate (11). However, Karimzade *et al* have shown that local injury to the endometrium on the day of oocyte retrieval disrupts the receptive endometrium and has a negative impact on the implantation and IVF outcomes (12). Increased macrophage inflammatory protein 1B (MIP-1B) expression could possibly serve for prediction of implantation competence (13, 14). Although several studies have investigated the effects of local endometrial injury on the pregnancy and success rates following IVF and ICSI, a limited number of studies have been conducted on its effect on the outcome of the IUI cycles (10-13). Thus, the present study aims to investigate the effects of local endometrial injury on the outcome of IUI cycles.

## Materials and methods

**Patients**

The present randomized clinical trial was performed in infertility clinic of Ghadir hospital affiliated to Shiraz University of Medical Sciences, Shiraz, Iran during a 17-month period from January 2011 to May 2012. The study was conducted on 18-40-year-old women who suffered from unexplained infertility, mild male factor, and mild endometriosis. 

In order to find the etiology of infertility, all the patients underwent semen analysis (for the partners), hormonal assay, including thyroid stimulating hormone (TSH), prolactin (to rule out hypophyseal adenomas), day 3 follicle-stimulating hormone (FSH), and day 3 luteinizing hormone(LH), hysterosalpingogram (HSG), laparoscopy, and hysteroscopy (to rule out uterine/tubal factors, including peritubular adhesions and endometriosis). All the women who had normal plasma concentrations of day 3 LH and FSH, normal tests of renal and hepatic function, normal complete blood counts, and negative pregnancy tests were enrolled into the study. On the other hand, the patients who, had hirsutism, autoimmune disorders, endocrinopathies, and ovarian hyperstimulation syndrome, smoked cigarettes, and abused alcohol (both partners) were excluded from the study. When the endometriosis was diagnosed, the stage was determined according to the revised Reproductive American Society for Medicine classification and the score was recorded (15). 

At least two semen analyses were performed for the men with male infertility. Normal semen analyses were defined by the threshold values of the World Health Organization (16); i.e., concentration of 15×10^6^/mL, total count of 39×10^6^, progressive motility of 32%, and typical morphology of 4%. Mild male factor subfertility was defined by the presence of at least one abnormal semen parameter; i.e., 5×10^6^> sperm count <15×10^6^/mL, total motility less than 40%, or the sperm's progressive motility of less than 32%. 

The study protocol was approved by the institutional review board (IRB) of Shiraz University of Medical Sciences, Shiraz, Iran and the approval of the Ethics Committee was achieved before beginning of the study. In addition, written informed consents were obtained from all the study participants.

**Study design and assays**

A total of 146 women were screened regarding eligibility for the study. These patients underwent 231 cycles of IUI. The patients were randomly divided into two groups through block randomization. Group A (n=74 IUI cycles=126) underwent endometrial biopsy at early follicular phase between days 6–8 of the menstrual cycle. On the other hand, group B (n=72, IUI cycles=105) underwent no intervention before the IUI cycles. 

Endometrial biopsy in group A patients was taken by a Novak curette biopsy catheter (Neuilly-en-Thelle, France) between the 6^th^ and the 8^th^ days of the menstrual cycle before the IUI treatment. Two small biopsies were obtained from anterior and posterior walls of the uterus. It should be mentioned that the patients were requested to use non-hormonal means of contraception during this cycle. 

All the patients received 100-mg per day clomiphene citrate (Razak Drug Laboratory, Tehran, Iran) for 5 days from the 5^th^ to the 9^th^ day of the cycle. Besides, 100 unit/day of highly purified recombinant FSH (Gonal-f, Serono, Hellas, Puregon, Greece) was injected subcutaneously from the 8^th^ day of the cycle. Transvaginal sonography was also performed on the 11^th^ day of the cycle and according to the size and number of the stimulated follicles, rFSH was continued until at least one less than 18mm dominant follicle was seen. 

Then, 10000 units of human chorionic gonadotropin (hCG) (Choriomon, IBSA, Switzerland) were injected intramuscularly if the serum E_2_ level was below 1500 pg/ml. The endometrial thickness (ET) was measured on the same day at its greatest diameter perpendicular to the midsagittal plane at the fundal region of the uterus.

IUI was performed 36 hours after hCG injection by two experts using a IUI catheter (BioRad, Berkeley, California). Sperm was prepared through the swim-up method (A method used to isolate motile spermatozoa from a washed sample of at least 10 million motile spermatozoa). β-hCG was checked if the patient experienced one week missed period. Moreover, pregnancy was documented by transvaginal sonography at 6-7 weeks of gestation. Each patient underwent up to three cycles of IUI. The main measurement outcomes were pregnancy rate per patient, pregnancy rate per cycle, abortion rate, multiple pregnancy rate, ongoing pregnancy rate (calculated by subtracting the abortion rate from the pregnancy rate), endometrial thickness, and number of the follicles larger than 18mm. 

**Statistical analysis**

Based on the power of 90% and α coefficient of 0.05 to detect significant differences between the two study groups regarding the pregnancy rate (p=0.05, 2-sided) and according to the previous report of endometrial biopsy for increasing the success rate in IVF, 72 cycles of IUI were needed in each study group (10). In order to compensate for non-evaluable patients, we included a total of 146 patients undergoing 231 IUI cycles. The statistical software package SPSS for Windows, version 16.0 (SPSS, Chicago, IL, USA) was used for data analysis. 

Paired *t*-test was used to compare the results within the groups, while independent t-test was utilized to compare the results between the groups. In addition, the proportions were compared using x^2^ test. The data were reported as mean±SD and p<0.05 was considered as statistically significant.

## Results

**Figure 1:**
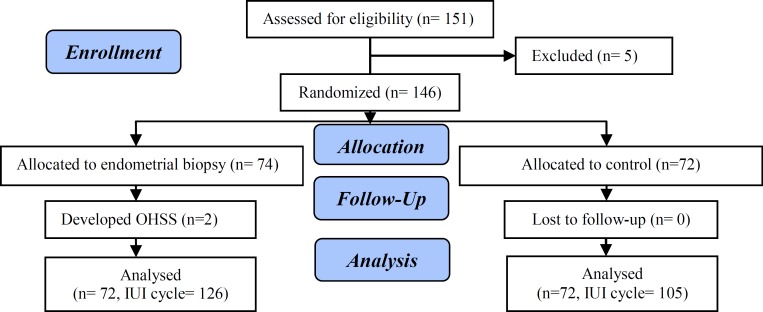
Flowchart of the study

Among the 151 eligible patients with unexplained infertility, mild male factor, and mild endometriosis, 5 were excluded from the study because of rejecting to participate the study. Thus, the remaining 146 patients were divided into two study groups; these patients underwent 231 cycles of IUI (126 in the endometrial biopsy group and 105 in the control group). During the study, 2 patients in endometrial biopsy group developed OHSS and were excluded from the study. Therefore, the final number of the patients and IUI cycles were 144 and 231, respectively (Figure 1). Table I summarizes the demographic and obstetric characteristics of the two study groups. The pregnancy rate per patient was found to be 17 (23.6%) and 14 (19.4%) in the endometrial biopsy and control groups, respectively. 

No statistically significant difference was found between the two study groups regarding the pregnancy rate per patient (OR=0.781, 95% CI: 0.352-1.734; p=0.686). The total pregnancy rate per cycle was 13.4% which was comparable between the two groups (13.5% vs. 13.3%; OR=0.846, 95% CI: 0.0.538-1.892; p=0.389) (Table II). The abortion rate was also comparable between the two study groups (6.9% vs. 9.7%; OR=1.443, 95% CI: 0.436-4.778; p=0.764). The ongoing pregnancy rate was found to be comparable between the two study groups, as well (16.7% vs. 9.7%; OR=0.538, 95% CI: 0.199-1.458; p=0.325).

**Table I:** Baseline characteristics of the patients in the two study groups

			**Endometrial biopsy group**	**Control group**	**p-value**
Age (years)*			28.1 ± 4.2	28.4 ± 5.1	0.767
Infertility duration (years)*			4.4 ± 3.1	5.6 ± 4.7	0.075
BMI (kg/m^2^) *			25.4 ± 3.6	25.1 ± 2.5	0.158
Infertility type**					0.186
		Primary (%)	56 (77.8%)	63 (87.5%)	
		Secondary (%)	16 (22.2%)	9 (12.5%)	
Infertility etiology**					
	Unexplained (%)		53 (73.6%)	46 (63.9%)	0.078
	Mild male factor (%)		16 (22.2%)	19 (26.4%)	0.328
	Mild endometriosis (%)		3 (4.2%)	7 (9.7%)	0.122
Day 3 LH (mIU/mL)*			5.4 ± 2.6	6.6 ± 6.4	0.164
Day 3 FSH (mIU/mL)*			6.2 ± 2.4	5.9 ± 2.5	0.463

N= 72

Student t-test

BMI: Body Mass Index

* are presented as mean±SD

** are expressed in frequency (%)

**Table II:** The outcomes of the IUI cycles in infertile patients undergoing endometrial biopsy and the controls

			**Endometrial biopsy group**	**Control group**	**p-value**
Number of IUI cycles**			126	105	
		1 cycle	38 (52.8%)	46 (63.9%)	0.067
		2 cycles	14 (19.4%)	19 (26.4%)	0.098
		3 cycles	20 (27.8%)	7 (9.7%)	0.020
Pregnancy rate per patient **			17 (23.6%)	14 (19.4%)	0.686
Pregnancy rate per cycle **			17/126 (13.5%)	14/105 (13.3%)	0.389
Abortion rate **			5 (6.9%)	7 (9.7%)	0.764
Ongoing pregnancy rate **			12 (16.7%)	7 (9.7%)	0.325
Pregnancy type**					
	Single		12 (16.7%)	6 (8.3%)	0.039
	Twin		0 (0.0%)	1 (1.4%)	0.042
E_2_ (pmol/L)*^, ^a			767.6 ± 532.2	1088.8 ± 545.1	<0.001
Endometrial thickness (mm)*			7.6 ± 1.2	7.7 ± 0.9	0.609

N= 72

* are presented as mean±SD

** are expressed in frequency (%)

a Chi-square test : on day of hCG administrationp<0.05 was considered statistically significant.

## Discussion

The current randomized clinical trial aimed to investigate the effects of endometrial injury on the outcome of the IUI cycles in those suffering from infertility. We found that in comparison to the control group, endometrial injury through endometrial biopsy within the follicular phase of the previous cycle of the IUI was associated with neither higher pregnancy rate per patient nor higher pregnancy rate per cycle following the IUI cycles. However, endometrial injury was associated with lower multiple pregnancy rate and lower serum E_2_ levels compared to the controls. These results indicate that endometrial injury prior to IUI by endometrial biopsy does not affect the outcome of the IUI cycles which is in contrast to some previous reports (10-14). 

Several previous studies have investigated the effects of endometrial injury on the outcome of ARTs, including IVF and ICSI, and reported contradictory results (10-14). For instance, Barash *et al* included a group of 134 patients, defined as good responders to hormonal stimulation, with repeated implantation failure (10). In that study, the IVF treatment and ET were preceded by repeated endometrial biopsies and the results showed that it doubled the chance for a take-home baby. In the same line, Zouh *et al* found that local injury to the endometrium during a COH cycle improved the rates of embryo implantation, clinical pregnancy, and live birth in ART (11). 

In a similar study, Gnainsky *et al *found that a biopsy-induced inflammatory response might facilitate the preparation of the endometrium for implantation (13). They suggested that increased MIP-1B expression could possibly serve for prediction of implantation competence. However, conflicting results were reported by Karimzade and colleagues (12). They evaluated the effect of local injury to the endometrium on the day of oocyte retrieval on implantation and pregnancy rates in assisted reproductive cycles. The results demonstrated that local injury to the endometrium on the day of oocyte retrieval disrupted the receptive endometrium and had a negative impact on implantation and IVF outcomes. Nevertheless, our study findings showed that the endometrial injury prior to the IUI cycles neither alleviated nor aggravated the pregnancy rate. 

One explanation for these findings could be the longer interval between the endometrial injury and IUI. We performed endometrial biopsies in the early follicular phase. In some previous studies, trauma to the endometrium was done in the luteal phase preceding the COH and there was sufficient time for gene expression, cytokine production, and other positive effects of the injury on the endometrium (10, 11). Thus, we think that there should be a shorter gap between endometrial biopsy and IUI or embryo transfer. 

Further work, including immunohistochemistry for endometrial biopsy, histologic observation, scanning electron microscopy (SEM) for pinopodes, molecular biology studies, and other experiments, are required to be done to explore this hypothesis. There are three possible mechanisms by which endometrial sampling may increase the receptivity and improve the clinical pregnancy rate of IVF-ET which may be effective in IUI cycles too. First, local injury to the endometrium might induce the decidualization of the endometrium and increase its implantation rate. Loeb reported that scratching guinea-pig uteruses provoked the rapid growth of the endometrial cells which are similar to the decidual cells of pregnancy (17). 

Second, local injury to the endometrium might provoke the wound healing process, involving a massive secretion of different cytokines and growth factors, including leukemia inhibitory factor, interleukin-11, and heparin-banding endothelial growth factor, which are beneficial for embryo implantation (18). The last and the most possible mechanism is COH performed during IVF therapy that may negatively affect the embryo implantation (19). Mirkin *et al* reported that COH cycles resulted in different structural and functional changes compared to the natural cycles (12). 

Our study had some limitations. First, all the patients in our study did not undergo equal and complete IUI cycles. Each patient was supposed to undergo up to three cycles of IUI until she became pregnant. However, some of our patients did not follow the protocol. This may be responsible for the low pregnancy rate in this study. Second, we did not measure the inflammatory markers of the endometrium; so that we cannot comment on the role of inflammation in implantation. Therefore, further studies are required to elucidate the effects of endometrial injury on the outcome of the IUI cycles.

## Footnote

***Registration ID in IRCT: IRCT2012070810210N1***

***This article extracted from M.D. thesis. (Ghazal Azadi) ***
